# P-1305. Evaluation of In Vitro Synergistic Activity of Antimicrobial Combinations against Carbapenem-Resistant Acinetobacter baumannii

**DOI:** 10.1093/ofid/ofaf695.1493

**Published:** 2026-01-11

**Authors:** Gaurav Vijay Salunke, Sanjay Biswas

**Affiliations:** Tata Memorial Hospital, Mumbai, Maharashtra, India; Tata Memorial Hospital, Mumbai, Maharashtra, India

## Abstract

**Background:**

Carbapenem-resistant *Acinetobacter baumannii* (CRAB) presents a significant healthcare concern owing to restricted treatment alternatives and elevated mortality rates. This study assesses the in vitro synergistic effects of antimicrobial combinations to determine appropriate treatment alternatives.

**Table 1:**
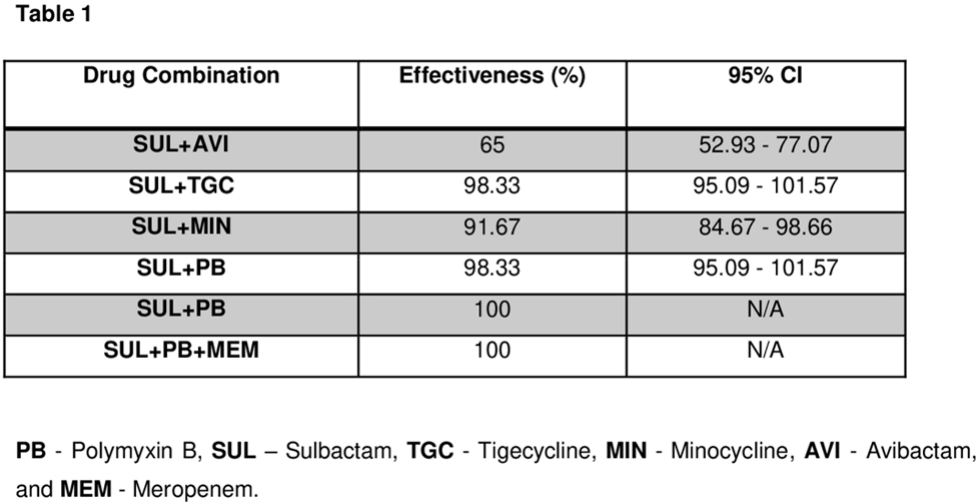
In Vitro Synergistic Activity of Antimicrobial Combinations

**Methods:**

Sixty non-duplicate CRAB isolates were identified and tested using the VITEK 2 system. Resistance mechanisms were determined via PCR for *blaNDM* and *blaOXA-48*. Synergistic activity of Polymyxin B (PB) + Sulbactam (SUL), Tigecycline (TGC) + SUL, Minocycline (MIN) + SUL, Sulbactam + Avibactam (SUL-AVI), and PB + Meropenem (MEM) + SUL was assessed using the Broth Disk Elution (BDE) method.

**Results:**

A total of 60 clinical isolates were tested for in vitro synergy using different antibiotic combinations. Effectiveness was defined as no bacterial growth (-) in the presence of a drug combination. The most effective combinations were SUL+PB and SUL+PB+MEM, showing 100% synergy (no bacterial growth). SUL+TGC and SUL+PB were highly effective (98.33%, CI: 95.09%-101.57%), followed by SUL+MIN (91.67%, CI: 84.67%-98.66%). SUL+AVI was the least effective (65%, CI: 52.93%-77.07%) [Table 1]. Chi-square analysis showed no significant difference between SUL+TGC, SUL+PB, and SUL+PB+MEM (p > 0.05). Advanced pairwise Z-tests also found no statistically significant differences among combinations (all p-values > 0.05).

**Conclusion:**

Polymyxin B-based regimens, particularly in combination with Meropenem and Sulbactum, appear highly promising for use in critically ill patients where rapid, broad-spectrum activity is paramount.

**Disclosures:**

All Authors: No reported disclosures

